# Coronary Plaque Characteristics and Cut-Off Stenosis for Developing Spasm in Patients with Vasospastic Angina

**DOI:** 10.1038/s41598-020-62670-z

**Published:** 2020-03-31

**Authors:** Sang-Ho Jo, Ju Ho Sim, Sang Hong Baek

**Affiliations:** 1 Division of Cardiology, Department of Internal Medicine, Hallym University Sacred Heart Hospital, Anyang-si, Gyeonggi-do Korea; 2 Graduate School of Public Health, Yonsei University, Seoul, South Korea; 3 Department of Cardiovascular Medicine, Seoul St. Mary’s Hospital, The Catholic University, of Korea, Seoul, South Korea

**Keywords:** Interventional cardiology, Ischaemia

## Abstract

Coronary plaque distribution, frequency and cut-off value of percent stenosis for developing vasospasm are uncertain in patients with vasospastic angina (VA). We enrolled 2960 patients who received coronary angiography (CAG) and ergonovine provocation test prospectively in 11 university hospitals in Korea. A total of 1836 patients with VA and 867 without VA were included. Plaque and % stenosis were defined as ≥1% luminal narrowing and mean of each segmental stenosis. Overall frequency of plaque and % diameter stenosis was compared among VA-patients with index coronary spasm positive, those with index arterial spasm negative/other arterial spasm positive (INOP) and non-VA patients. Diameter stenosis associated with the spasm positivity was investigated. Overall plaque frequency and % stenosis were higher in VA patients than non-VA patients. Plaque frequency was 27.6% (243/881) in spasm positive at LAD, 16.4% (157/955) in LAD INOP and 12.6% (109/867) in non-VA with statistic difference (P < 0.001). Same trend for higher rate was observed in LCx and RCA. For % stenosis, 36.6 vs 32.4% (p = 0.010) in LAD, 36.1 vs. 28% (p < 0.001) in LCx and 35.3 vs.30.0% (p = 0.047) in RCA, respectively. Diameter stenosis of LAD with spasm positive vs. LAD INOP vs. non-VA were 38.3%, 34.0%, 32% (P = 0.002) with similar pattern in LCx and RCA. By multivariate logistic regression analysis, coronary stenosis of LAD ≥ 35% or LCx ≥35% or RCA ≥ 40% were independent predictor of developing spasm (OR 2.019, 95% CI 1.315–3.100, P = 0.001). In conclusions, spastic coronary artery had more plaque frequency, higher % stenosis than in non-spastic coronary in VA patients. The spasm related and unrelated coronary in VA patients had more plaque than in matched and unmatched coronary arteries in non-VA patients. Coronary stenosis ≥35% in LAD and LCx was an independent predictor of developing spasm.

## Introduction

Vasospasm usually occurs at the site of coronary plaque^1–3^ and even minimal atherosclerotic plaque on which the spasm occurred is reported to be associated with poor prognosis^4^. Vascular endothelial dysfunction and hyper-reactive vascular smooth muscle (VSM) at the atherosclerotic site could explain the link between vasospasm and atherosclerosis^2,5^. Nonetheless, fundamental data regarding the atherosclerotic plaque like frequency, burden, location and distribution in coronary artery at which the spasm occurs has never been performed. The overall atherosclerosis status in non spastic artery of vasospastic angina (VA) patients has also never been investigated which could help, at least in part, elucidate the true association of atherosclerosis and vasospasm or causal relationships.

We investigated as well, the cut-off vales of atherosclerotic diameter stenosis of coronary artery which are linked with provoking vasospasm. These works are meaningful because the findings could contribute to management of VA patients and develop other therapeutic measures like lipid or atherosclerosis modifying manipulation for preventing spasm as well as to help more understand pathophysiology of VA.

We investigated for the first time, the association of vasospasm and atherosclerotic plaque in detail with a large scale prospective registry of VA- patients form Korea.

## Results

**Figure 1:**
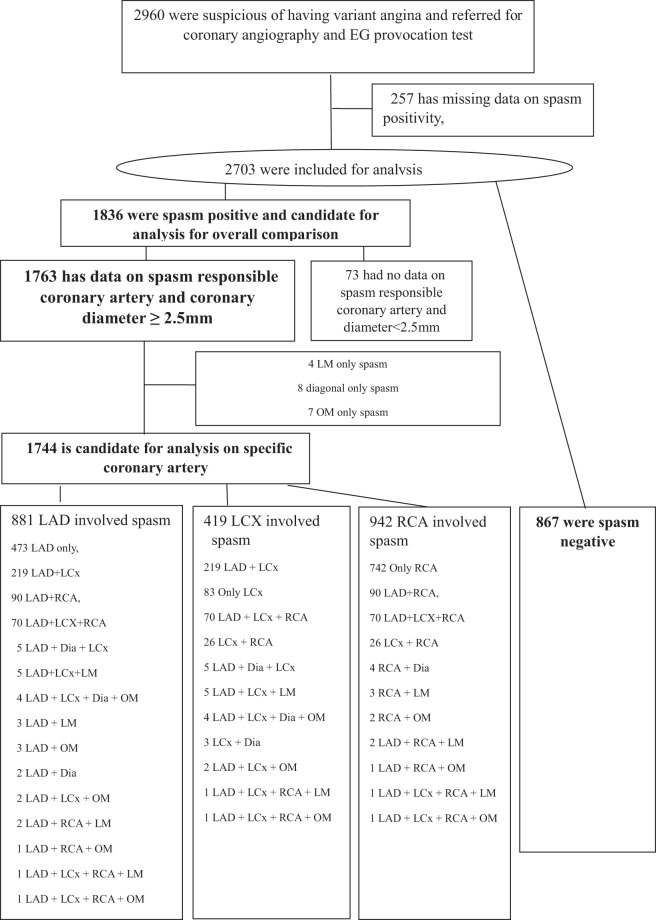
Study flow. Abbreviations: LM, left main coronary artery; OM, obtuse marginal branch; LAD, left anterior descending coronary artery; LCx, left circumflex coronary artery; RCA, right coronary artery; Dia, diagonal branch.

A total of 1836 patients with VA and 867 with non-VA are included for overall comparison for their atherosclerosis status (Fig. 1). Baseline characteristics between VA and non-VA patients were comparable except for male sex, active smoker, percutaneous coronary intervention (PCI) history, high density lipoprotein cholesterol (HDL-C), high-sensitivity C-reactive protein (hsCRP), Troponin (Table 1). After 73 patients who do not have specific spasm location data with coronary diameter <2.5 mm were excluded in specific coronary focusing comparison, 1763 patients was included in spasm location oriented comparison with INOP and non-VA patients (Fig. 1).

**Table 1:** Baseline characteristics of spasm positive and negative groups.

	Spasm positive (n = 1836)	Spasm negative (n = 867)	p value
Gender			
Male	1,141 (62.1)	336 (38.8)	<0.001
Female	695 (37.9)	531 (61.2)	
Age (years) (Mean ± SD)	55.1 ± 11.3	54.7 ± 13.0	0.454
BMI (kg/m^2^) (Mean ± SD) *	24.8 ± 3.6	24.7 ± 3.4	0.552
Hypertension			
Yes	694 (37.8)	307 (35.4)	0.226
No	1,141 (62.2)	560 (64.6)	
Diabetes			
Yes	175 (9.5)	86 (9.9)	0.760
No	1,658 (90.5)	781 (90.1)	
Dyslipidemia			
Yes	299 (16.3)	145 (16.7)	0.805
No	1,530 (83.7)	722 (83.3)	
Smoking			
Active smoker	501 (27.7)	118 (13.8)	<0.001
Ex-smoker & Never	1,308 (72.3)	738 (86.2)	
Hx of CAD			
Yes	219 (11.9)	90 (10.4)	0.238
No	1,617 (88.1)	777 (89.6)	
Angina with evidence of IHD			
Yes	73 (4.0)	44 (5.1)	0.190
No	1,763 (96.0)	823 (94.9)	
CAD with medical Treatment after coronary angiography			
Yes	106 (5.8)	37 (4.3)	0.103
No	1,730 (94.2)	830 (95.7)	
CAD with PCI			
Yes	40 (2.2)	9 (1.0)	0.038
No	1,796 (97.8)	858 (99.0)	
CAD with CABG			
Yes	0 (0.0)	0 (0.0)	—
No	1,836 (100.0)	867 (100.0)	
Stroke			
Yes	31 (1.7)	19 (2.2)	0.370
No	1,800 (98.3)	848 (97.8)	
Transient ischemic attack			
Yes	7 (0.4)	2 (0.2)	0.524
No	1,822 (99.6)	864 (99.8)	
Chronic kidney disease			
Yes	5 (0.3)	5 (0.6)	0.226
No	1,826 (99.7)	862 (99.4)	
Family Hx of CAD			
Yes	98 (5.3)	61 (7.0)	0.081
No	1,734 (94.7)	805 (93.0)	
Hx of calcium channel blocker			
Yes	352 (19.4)	160 (18.5)	0.591
No	1,461 (80.6)	703 (81.5)	
Hx of aspirin			
Yes	366 (20.1)	161 (18.6)	0.358
No	1,455 (79.9)	705 (81.4)	
Hx of thienopyridine			
Yes	69 (3.8)	22 (2.5)	0.093
No	1,744 (96.2)	842 (97.5)	
Hx of statin			
Yes	282 (15.6)	148 (17.1)	0.325
No	1,520 (84.4)	715 (82.9)	
Hx of ARB or ACEI			
Yes	318 (17.6)	144 (16.7)	0.552
No	1,487 (82.4)	719 (83.3)	
Hx of beta-blocker			
Yes	137 (7.6)	77 (8.9)	0.246
No	1,666 (92.4)	788 (91.1)	
LDL-C (mg/dL) (Mean ± SEM)	103.6 ± 0.8	105.9 ± 1.2	0.098
HDL-C (mg/dL) (Mean ± SD)	46.7 ± 12.9	48.0 ± 12.5	0.010*
hs-CRP (mg/dL) (Mean ± SD)	0.9 ± 6.7	0.6 ± 3.1	0.042^§^
Troponin (Mean ± SD)	0.61 ± 4.52	0.3 ± 1.9	0.044^§^
CK-MB (Mean ± SEM)	5.9 ± 0.6	4.0 ± 0.4	0.168^*^
LVEF (%) (Mean ± SD)	64.4 ± 6.7	64.6 ± 6.2	0.700

Values are expressed as mean ± standard deviation (SD) or mean ± standard error of the mean (SEM).

SD, standard deviation; BMI, body mass index; Hx, history; CAD, coronary artery disease; IHD, ischemic heart disease; PCI, percutaneous coronary intervention; CABG, coronary arterial bypass graft; ARB, angiotensin receptor blocker; ACEI, angiotensin converting enzyme inhibitor; LDL-C, low density lipoprotein cholesterol; SEM, standard error of the mean; HDL-C, high density lipoprotein cholesterol; hs-CRP, high-sensitivity C-reactive protein; CK-MB, creatine-kinase MB; LVEF, left ventricular ejection fraction.

*Log

^§^Mann-Whitney U test.

BMI: 105 cases missing in spasm positive and 59 cases missing in spasm negative.

Hypertension: 1 case missing in spasm positive.

Diabetes: 3 cases missing in spasm positive.

Dyslipidemia: 7 cases missing in spasm positive.

Smoking: 27 cases missing in spasm positive and 11 cases missing in spasm negative.

Stroke: 5 cases missing in spasm positive.

Transient ischemic attack: 7 cases missing in spasm positive and 1 case missing in spasm negative.

Chronic kidney disease: 5 cases missing in spasm positive.

Family Hx of CAD: 4 cases missing in spasm positive and 1 case missing in spasm negative.

Hx of calcium channel blocker: 23 cases missing in spasm positive and 4 cases missing in spasm negative.

Hx of aspirin: 15 cases missing in spasm positive and 1 case missing in spasm negative.

Hx of thienopyridine: 23 cases missing in spasm positive and 2 cases missing in spasm negative.

Hx of Statin: 34 cases missing in spasm positive and 4 cases missing in spasm negative.

Hx of ARB or ACEI: 31 cases missing in spasm positive and 4 cases missing in spasm negative.

Hx of beta-blocker: 33 cases missing in spasm positive and 2 cases missing in spasm negative.

### Overall distribution and burden of coronary atherosclerotic plaque in VA patients vs. Non-VA patients

**Figure 2:**
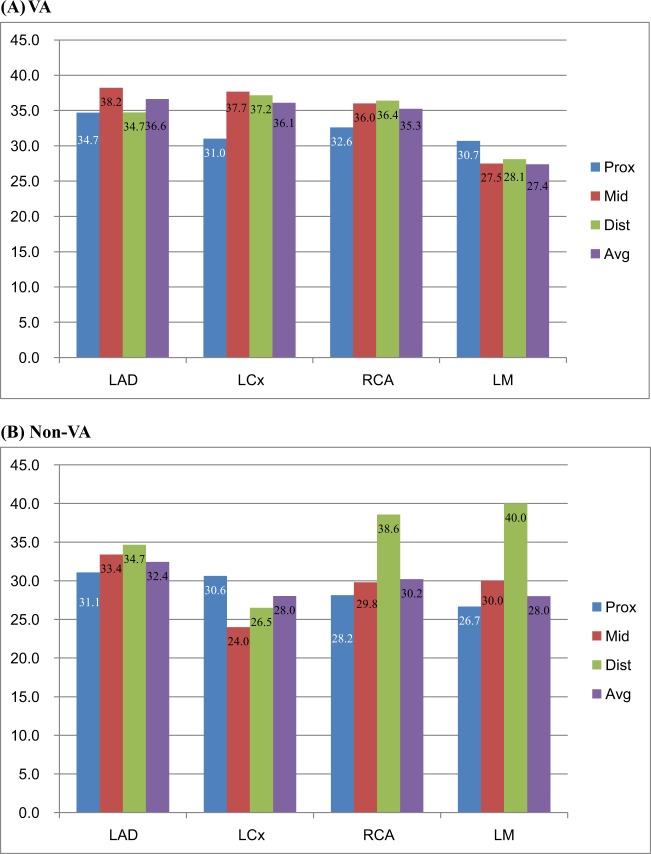
Coronary diameter percent stenosis of each coronary segment in VA and non-VA groups. Abbreviations: VA, variant angina; LAD, left anterior descending coronary artery; LCx, left circumflex coronary artery; RCA, right coronary artery; LM, left main coronary artery; Prox, proximal segment; Mid, middle segment; Dist, distal segment; AVG, average (mean value of each segment % stenosis).

The atherosclerotic coronary artery which is defined as having at least one plaque at any coronary artery was more common in VA patients - irrespective of spasm location- than in non-VA patients with LAD, 22.5% (414/1836) vs. 12.6% (109/867) (p < 0.001) in left anterior descending coronary artery (LAD), 9.4% (173/1863) vs. 3.2% (28/867) (p < 0.001) in left circumflex artery (LCx) and 16.8% (308/1836) vs. 6.6% (57/867) (p < 0.001) in right coronary artery (RCA) respectively. (Table 2) Diameter percent stenosis was also higher in VA group than in non-VA group, 36.6 ± 16.6 vs. 32.4 ± 14.2% (p = 0.010) in LAD, 36.1 ± 18.3 vs. 28.0 ± 8.2% (p < 0.001) in LCx and 35.3 ± 17.5 vs.30.2 ± 15.5% (p = 0.047) in RCA respectively (Table 3 and Fig. 2)

**Table 2:** Plaque frequency of each coronary artery with atherosclerosis between spasm positive and negative patients (lesion overlapping permitted).

Plaque location (N)	Spasm Positive and Plaque Positive Patients, n (%)*	Spasm Negative and Plaque Positive Patients, n (%)*	p-value
LM (24)	19 (1.0)	5 (0.6)	0.236
LAD (523)	414 (22.5)	109 (12.6)	<0.001
Diagonal (60)	53 (2.9)	7 (0.8)	<0.001
LCx (201)	173 (9.4)	28 (3.2)	<0.001
OM (19)	17 (0.9)	2 (0.2)	0.043
RCA (365)	308 (16.8)	57 (6.6)	<0.001

*% in (A) panel indicates 100 x ratio of plaque positive patients/total patients in VA and non-VA group respectively (i.e. 1836 and 867 respectively in each group).

LM, left main coronary artery; LAD, left anterior descending coronary artery; LCx, left circumflex coronary artery; OM, obtuse marginal branch; RCA, right coronary artery; SD, standard deviation.

**Table 3:** Mean percent stenosis of each coronary artery with atherosclerosis between spasm positive and negative patients (lesion overlapping permitted).

Plaque location (N)	Spasm Positive and Plaque Positive (N = 984) mean ± SD (n)	Spasm Negative and Plaque Positive (N = 208) mean ± SD (n)	p-value
LM (24)	27.4 ± 6.3 (19)	28.0 ± 14.8 (5)	0.930
LAD (523)	36.6 ± 16.6 (414)	32.4 ± 14.2 (109)	0.010
Diagonal (60)	46.1 ± 23.0 (53)	58.6 ± 23.4 (7)	0.183
LCx (201)	36.1 ± 18.3 (173)	28.0 ± 8.2 (28)	<0.001
OM (19)	45.4 ± 21.8 (17)	58.3 ± 38.8 (2)	0.681
RCA (365)	35.3 ± 17.5 (308)	30.2 ± 15.5 (57)	0.047

Values are expressed as mean ± standard deviation.

LM, left main coronary artery; LAD, left anterior descending coronary artery; LCx, left circumflex coronary artery; OM, obtuse marginal branch; RCA, right coronary artery; SD, standard deviation.

**Figure 3:**
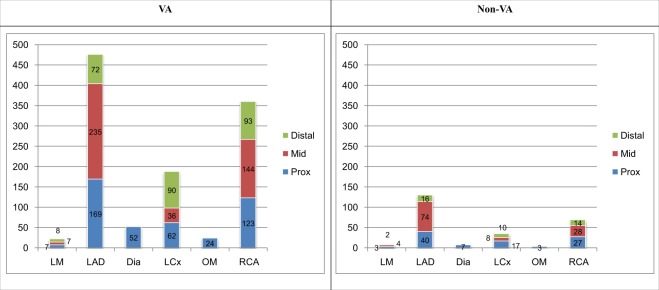
Atherosclerotic plaque distribution in each coronary segment (lesion based) in VA and non-VA patients groups. Vertical axis indicates plaque number. Numbers in graph indicate lesion count in each segment. Abbreviations: VA, variant angina; LM, left main coronary artery; LAD, left anterior descending coronary artery; Dia, diagonal branch; LCx, left circumflex coronary artery; OM, obtuse marginal branch; RCA, right coronary artery; Distal, distal segment; Mid, middle segment; Prox, proximal segment.

The detailed segmental distribution of plaque was presented in Fig. 3. Segmental distribution of atherosclerotic plaque was more prevalent in spastic patients. (Fig. 3)

### Atherosclerosis analysis focusing on individual coronary: spasm positive at index coronary artery vs. index coronary spasm negative but positive on other coronary (INOP) vs. Non-VA patients

Diameter % stenosis is higher in VA patients than in non-VA patients at each coronary artery, 38.27 ± 16.54% vs. 33.36 ± 15.58% (p = 0.001) in LAD, 40.15 ± 19.64% vs. 32.07 ± 15.47 in LCx (p = 0.005) and 35.84 ± 17.83% vs. 32.44 ± 16.33% in RCA (p = 0.07) (Fig. 3).

VA-patients was always more likely to have more atherosclerosis at spastic coronary than the other coronary within same VA-patients (Tables 4, 5 and Supplementary Table 1). For example, LAD spasm positive patients had higher plaque frequency in LAD (27.6% [LAD], 8.1% [LCX], 10.2% [RCA]) (Table 4), higher mean % stenosis in LAD (38.3% [LAD], 32.5% [LCX], 33.2% [RCA]) (Table 5) and higher plaque burden in LAD (45.2% [LAD], 39.8% [LCX], 39. 9% [RCA]) than LCx and RCA. (Supplementary Table 1, vertical comparison). Patients with spasm positive at LCx and RCA also showed same trend, in plaque status like frequency, % stenosis and burden (Supplementary Table 1, vertical comparison)

**Table 4:** Plaque frequency of 3 coronary arteries in specific coronary artery (≥2.5 mm diameter) in spasm positive patients focused view point.

Plaque location (n)	LAD spasm positive (N = 881), n (%)	LCx spasm positive (N = 419), n (%)	RCA spasm positive (N = 942), n (%)
LAD plaque yes	**243 (27.6)**	73 (17.4)	175 (18.6)
LAD plaque no	638 (72.4)	346 (82.6)	767 (81.4)
LCx plaque yes	71 (8.1)	**68 (16.2)**	74 (7.9)
LCx plaque no	810 (91.9)	351 (83.8)	868 (92.1)
RCA plaque yes	90 (10.2)	46 (11.0)	**213 (22.6)**
RCA plaque no	791 (89.8)	373 (89.0)	729 (77.4)

LAD, left anterior descending coronary artery; LCx, left circumflex coronary artery; RCA, right coronary artery.

**Table 5:** Mean diameter percent stenosis in 3 coronary arteries in specific coronary artery (≥2.5 mm diameter) spasm positive patients focused view point.

Stenosis location	LAD spasm positive with plaque	LCx spasm positive with plaque	RCA spasm positive with plaque
LAD stenosis, % (n)	**38.3** ± **16.5 (243)**	36.0 ± 15.0 (73)	33.9 ± 16.4 (175)
LCx stenosis, % (n)	32.5 ± 13.2 (71)	**40.2** ± **19.6 (68)**	33.9 ± 16.4 (74)
RCA stenosis, % (n)	33.2 ± 17.4 (90)	35.3 ± 18.5 (46)	**35.8** ± **17.8 (213)**

Values are expressed as mean ± standard deviation.

LAD, left anterior descending coronary artery; LCx, left circumflex coronary artery; RCA, right coronary artery.

**Figure 4:**
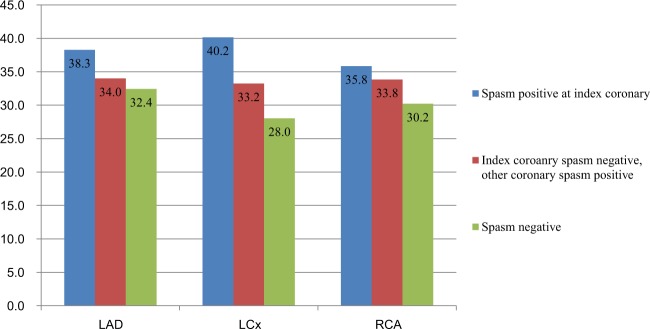
Coronary diameter percent stenosis of index coronary spasm positive vs. index negative but other positive vs. spasm negative patients group in each coronary. Vertical axis indicates % stenosis. LAD, left anterior descending coronary artery; LCx, left circumflex coronary artery; RCA, right coronary artery.

In comparing specific spastic coronary artery in VA-patients with those from INOP and non-VA patients, plaque parameter (plaque frequency, % stenosis, burden) linearly decreased in order of highest in patients with index coronary spasm positive on which the spasm occurred, middle in INOP and lowest (or similar to INOP) in non-VA patients with statistic differences (Fig. 4, Supplementary Table 1, horizontal comparison).

In LAD spasm positive patients, LAD plaque frequency were 27.6%, 16.4%, 12.2% in that order respectively (p < 0.001). In LAD spasm positive patients, LAD plaque diameter stenosis were 38.27 ± 16.54%, 33.99 ± 16.50, 32.43 ± 14.15 with p = 0.002 (highest in LAD coronary spasm positive on which the spasm occurred than INOP and non-VA. INOP and non-VA is similar) (Supplementary Table 1). Plaque burden followed same trend in LAD (Supplementary Table 1).

In case of LCx and RCA spasm, there was a trend for highest plaque parameter in index coronary spasm positive than INOP and non-VA (with INOP ≥ non-VA). (Supplementary Table 1).

Even the spasm irrelevant coronary in VA patients are more likely to have higher plaque status parameters than those matching coronary with non-VA patients. For example, in LAD spasm positive case, LCx plaque frequency was highest in LAD spasm case (8.1%) than that of INOP (9.9%) and non-VA (3.2%) (Supplementary Table 1). Same trend was observed in plaque stenosis and burden (Supplementary Table 1).

### Finding diameter % stenosis cut-off value determining spasm positivity

Mean lumen narrowing for determining LAD spasm was ≥35% of diameter stenosis with 47.7% sensitivity and 66.4% specificity (Odds ratio [OR] = 1.806, 95% confidence interval [CI] 1.261–2.587, P = 0.001). Other criteria in LAD like diameter stenosis ≥ 30% has highest sensitivity of 79.4% with lowest specificity of 32.1% (OR 1.822, 95% CI 1.215–2.731, P = 0.004). Using LAD ≥ 60% of mean stenosis, we can get a highest specificity of 93.5% with lowest sensitivity of 12.8% (OR 2.107, 95% CI 1.134–3.915, p = 0.018). (Supplementary Table 2). For LCx, ≥ 35% of mean % stenosis was ideal for detecting probable spastic coronary artery with 52.9% sensitivity and 71.2% specificity (OR 2.781, CI 1.506–5.138, P = 0.001) and for RCA ≥ 40% stenosis had 36.6% sensitivity and 73.4% specificity with OR 1.596 (95% CI 1.004–2.1539, P = 0.048). (Supplementary Table 2).

Multivariate logistic regression analysis had provided that LAD stenosis ≥ 35% (OR 2.434, 95% CI 1.40–4.232, P = 0.002) was an independent risk factors for determining vasospasm of LAD as well as male sex (OR 2.455, 95% CI 1.376–4.378, P = 0.002), age (negative risk, OR 0.974, 95% CI 0.950–0.998, P = 0.037 and smoking (OR 2.493, 95% CI 1.067–5.822, P = 0.035) (Supplementary Table 3).

For LCx, % stenosis of ≥35% were also an independent risk factor for determining LCx vasospasm (OR 8.349, 95% CI 2.086–33.412, P = 0.003) along with male sex (OR 3.756, 95% CI 1.057–13.347, P = 0.041) (Supplementary Table 3), but RCA ≥ 40% was not (OR 1.647, 95% CI 0.781–3.472, P = 0.190). When we combined the 3 vessels’ criteria of LAD stenosis ≥ 35% or LCx ≥ 35% or RCA ≥ 40%, this parameter was also an independent risk for spasm positive response by multiple logistic regression analysis (OR 2.019, 95% CI 1.315–3.100, P = 0.001) (Table 6).

**Table 6:** Combined coronary % stenosis with either LAD ≥35% or LCx ≥35% or RCA ≥40% for determining coronary arterial spasm positivity.

		Spasm Negative (N = 867)	Spasm Positive (N = 1744)	P-value	OR	95% CI	P-value
Gender	Female	531 (61.2)	664 (38.1)	<0.001	1		
	Male	336 (38.8)	1,080 (61.9)		3.197	2.035–5.022	**<0.001**
Age		54.8 ± 13.0	55.0 ± 11.4	0.575	0.990	0.970–1.010	0.336
BMI(kg/m^2^)		24.7 ± 3.4	24.8 ± 3.6	0.589	0.992	0.939–1.048	0.776
Hypertension	No	560 (64.6)	1,077 (61.8)	0.163	1		
	Yes	307 (35.4)	666 (38.2)		1.117	0.715–1.745	0.627
Diabetes	No	781 (90.1)	1,575 (90.5)	0.754	1		
	Yes	86 (9.9)	166 (9.5)		0.487	0.276–0.858	**0.013**
Dyslipidemia	No	722 (83.3)	1,448 (83.4)	0.956	1		
	Yes	145 (16.7)	289 (16.6)		0.759	0.435–1.325	0.332
Smoking*	No	738 (86.2)	1,239 (71.8)	<0.001	1		
	Yes	118 (13.8)	487 (28.2)		2.337	1.197–4.562	**0.013**
Hx of calcium-channel blocker^#^	No	703 (81.5)	1,384 (80.4)	0.508	1		
	Yes	160 (18.5)	338 (19.6)		0.853	0.519–1.401	0.530
LAD or LCx or RCA stenosis	Under Cut off	90 (66.7)	305 (52.7)	0.003	1		
	Over Cut off	45 (33.3)	274 (47.3)		2.019	1.315–3.100	**0.001**

*No: never & Ex-smoker / Yes: Active-smoker.

^#^11 cases in spasm positive, 18 cases in spasm positive had missing value.

OR, odds ratio; CI, confidence interval; SD, standard deviation; BMI, body mass index; Hx, history; LAD, left anterior descending coronary artery; LCx, left circumflex coronary artery; RCA, right coronary artery.

## Discussions

We investigated for the first time the detailed distribution, severity of atherosclerotic plaque and their relationship in VA patient as compared to non-VA patients. We found that the VA patients who has spasm at any coronary artery were more likely to have overall atherosclerotic plaque in all coronary arteries even in non-spastic artery as compared to non-VA patients. Spastic coronary has more likely to have plaque, higher plaque % stenosis and larger plaque burden than other coronary artery irrelevant to spasm in same VA patient. The plaque parameters were higher in VA patient both in spastic index artery and other coronary artery irrelevant to spasm than those with non-VA patients. These results are in line with those from previous small sized intravascular ultrasound (IVUS) study^6^ which is only one literature regarding this kind of subject.

We analyzed the plaque with more focus on specific spasm positive coronary artery to compare in-depth with other non-spasm responsible coronary of VA patient as well as coronaries of non-VA patients. Spasm positive coronary had more chance to have plaque, higher diameter stenosis and higher plaque burden as compared to matching coronary artery of non-VA patients. Interesting finding is that, in our combined comparing with those with index coronary not spastic but other site positive (INOP), the likelihood of having plaque, % stenosis and atheroma burden was highest in index arterial spasm positive, middle in INOP and lowest in non-VA patients. Same trend was observed in all 3 coronary arteries. Even the plaque in INOP showed higher or higher trend than that of non-VA patients consistently.

We also, for the first time, investigated the cut-off point of coronary stenosis which might be related to spasm provocation as 35–40% and found this value was independent predictor for spasm.

Our study results imply that the spasm is very closely linked with plaque. Spasm status might be associated with the whole vascular atherosclerotic circumstance even in non-spasm related artery as well as spasm culprit artery. This could reflect the systemic nature of vasospasm which is linked with atherosclerosis.

The concept that plaque can be a trigger for vasospasm or reversely, spasm itself can initiate or aggravate the atherosclerosis is already well known by experimental and clinical studies^7^. The spasm itself could cause atherosclerotic progression, plaque injury or rupture and could even lead to ST-elevation myocardial infarction from clinical or experimental evidences^1,8–11^. It is postulated that the mechanical insult by spasm on the coronary might have some role. Another similar mechanism is myocardial bridging which can cause a vascular event by repetitive mechanical insult to coronary artery. This coincided well with the hypothesis that vasospasm could induce mechanical injury of coronary artery even resulting in acute thrombotic events^12^.

On the contrary, atherosclerotic plaque can induce and aggravate vasospasm mainly driven by endothelial dysfunction, nitric oxide (NO) deficiency and mild inflammation of atherosclerosis and vascular smooth muscle cell (VSM) hyper-reactivity^13^. One IVUS–virtual histology study found that coronary segments with endothelial dysfunction had larger necrotic core plaques and the size of necrotic core plaques was the principal determinant of coronary endothelial dysfunction^14^. However the causal relationship of which one between spasm and atheroma is initiator has been uncertain.

Recent study arguing perivascular fat has a role in spasm also support the atherosclerosis could influence the spasm by VSM contraction pathway^15^. But we consider more important factors for spasm is intra-lumen plaque which could more directly affect the luminal circumstances as resulting in flow limitation and obstruction. Moreover peri-vascular adipose tissue could just be a reflection of vascular intimal atherosclerosis^16^. Thus, we think, intramural and intraluminal atherosclerosis might be more critical as our study results. We can also postulate that in view of the fact that the plaque burden is prevalent and higher in VA patients even in the spasm unrelated artery than that of non-VA patients, the atherosclerosis might have initiate and precipitate the vasospasm. In other words, the atherogenic milieu first and later on, the spasm occurred at site of minimal stenosis around 35–40% of lumen narrowing of coronary diameter, with moderate endothelial dysfunction and combined VSM hyper-reactivity. This concept is partly supported by very few evidence yet, and need to be further investigated^17^.

A study reported that VA patients with spasm at the site of plaque have poorer clinical outcomes than those with spasm at other site^18^. In that study, the authors defined the plaque as 75% or more diameter stenosis which is usually accepted cut-off vale of traditional atherosclerotic stenosis. However this value may not be true in the vasospasm, rather less luminal narrowing would be appropriate because VA patients could experience more severe stenosis enough to make flow limitation with vascular contraction on top of mild fixed lesion^19^. A research indicating even non-obstructive coronary artery disease (CAD) among non-VA population with angiographic stenosis 20–70% has nearly 3-fold increased risk of death compared with those without apparent CAD also support our hypothesis^20^. Thus less than 75% of spasm related atherosclerotic stenosis might be appropriate in spasm occurrence and determining prognosis. With this curiosity, we firstly aim to pursue the true cut-off point of diameter stenosis of spasm provocation in VA suspicion patients, and we are preparing for the cut-off diameter stenosis for determining poor clinical outcome in VA-patients.

In another view point of the plaque burden, we could postulate that much plaque burden could not provoke the spasm because the hyperactive vascular response hardly occurred due to lack of remaining endothelial dysfunction to exert spasm and few residual functional vascular smooth muscle cell in arterial media nor the vasoconstriction could not overcome the coronary arterial stiffness and volume of the plaque. Thus, we hypothesized that diameter stenosis which is related to development of vasospasm would be around 30–70% arbitrarily. With this assumption we tested the spasm prone cut-off % stenosis in each coronary artery and found appropriate value of ≥35% both in LAD and LCx and ≥40% in RCA which were remained meaningful by multivariate logistic regression analysis despite of little sensitivity. We assume that with this cut-off value, both VSM dysfunction and endothelial dysfunction start exerting effect on the vascular spasm.

### Clinical implication

The disease entity of VA is still remained to be elucidated. Some researchers suggested genetic background is closely associated with VA occurrence. For example, a person with lack of activity of aldehyde dehydrogenase (ALDH) 2 owing to having variant ALDH2*2 genotype which are related with alcoholic flushing syndrome are prone to suffer from VA^21^. Among lots of plausible mechanism and explanation for developing VA, we concentrate on the relationship between atherosclerosis and VA.

Our results could imply that the atherosclerotic plaque occupying around 1/3 of lumen could predict the spasm positive in VA suspicion patients. This could be of value in clinical practice for our assessing and predicting spasm positivity in ergonovine (EG) provocation test especially in case of the EG provocation response is obscure. We could also be more alert in those patients and may pay more attention to than those who have plaque less than 30–40% especially in case of uncertain EG provocation test results. In treatment view point, the atherosclerosis involved spasm positive patients ought to be candidate for statin therapy despite of the uncertainty of statin’s role in VA, because there is still possibility of minimal atherosclerotic plaque could have contribute spasm initiation or intensification from indirect data from our study. The role of statin in patients with plaque narrowing ≥ 35–40% of lumen ought to be further investigated in future study. Further research is on-going as to whether this value could predict prognosis of VA patients by our study group.

### Study Strength

Our study has some strength.

Firstly, we used the provocation methods with EG other than acetylcholine (ACH). Although both agents are useful and widely utilized in clinical practice, the EG might be better because it provokes vasospasm via VSM contraction not by endothelial mediated vasoconstriction which could be induced by ACH. The endothelial dysfunction could be usually revealed by ACH provocation test at early stage of atherosclerosis^22^, so the spasm could not occur in moderate to severe atherosclerotic artery. Moreover, some study argues that the endothelial dysfunction is not main mechanism, instead VSM hyper-constriction by RHO kinase activation is a major player^23,24^.

Secondly, we firstly evaluated the association of atheroma in coronary artery with spasm in detail using large scale prospective multicenter data and found the cut-off values of spasm inducing atheroma burden in VA patients with atheroma raging 35–40% which is lower what has been considered to be significant in general atherosclerosis patients.

Thirdly, we assessed the plaque in detail with 3 aspects like presence of plaque, dimeter % stenosis of coronary lumen and plaque burden expressed as summation of each segmental % stenosis and showed consistent results in all aspect of comparisons.

### Limitations

Firstly, our study was performed using registry data which has inevitable biases. For example, in our registry, patients who complain of chest pain suspicious of VA received coronary angiography and ergonovine provocation test at the physician’s discretion. Thus in some patients, the final diagnosis was not VA. Accordingly, selection bias can exist because the non-VA patients might have other hidden morbidity that can provoke chest pain other than coronary spasm, i.e. they could not have definitely normal coronary artery. However our registry is large scale, prospective and multicenter one that has value.

Secondly, the percent stenosis and burden of plaque was just estimated by visual and QCA not by IVUS or OCT which are more correct. Thus the measured value was expressed with unit of number 5. We also do not measure correct plaque volume, instead we employed the indirect methods of diameter stenosis and segmental stenosis summation. Nevertheless we perform QCA by independent person who are blind to the study performance in central laboratory facilities.

Thirdly, the cut-off value for sensitivity and specificity of lumen stenosis is low.

Fourthly, the rate of patients with hypertension, diabetes and chronic kidney disease are lower than other study. We consider this could be attributed to selection bias, recall bias or different population enrolled with only suspicious of VA which might have a little risk factors than those in other clinical studies.

## Methods

### Study objective

We aim to investigate the nature of atherosclerosis in VA patients like plaque frequency, distribution, burden and compare these with those of non-VA patients and index coronary arterial negative but other site coronary positive VA patients in detail. The final objective of the study is to find the insight of association or causal relationship between spasm and atherosclerosis in VA patients.

Study protocol was approved by ethics committees/institutional review board of each participating hospital including Hallym University Sacred Heart Hospital Institutional Review Board, named ‘HUMC ethics committees & IRB’ (IRB No. 2010-I007) and all patients gave written informed consent. All procedures performed in studies involving human participants were in accordance with the ethical standards of the institutional and/or national research committee and with the 1964 Helsinki declaration and its later amendments or comparable ethical standards.

### Patients

VA-Korea (Variant angina Korea) registry is a nation-wide prospective multicenter registry which enrolled patients with chest pain suspicious of VA who received coronary angiography (CAG) and EG provocation test^25,26^. Suspicion of VA was solely at a physician’s discretion. Hence patents with normal or atherosclerotic coronary artery can be included in this registry other than those with VA. Adults aged 18 or over were candidate for enrollment. Patients having malignancy, end stage renal disease on dialysis, inflammatory disease and catheter-induce spasm at baseline CAG were excluded. Hypertension was defined as either blood pressure ≥140/90 mmHg or the current use of anti-hypertensive medications. Diabetes was defined as having fasting glucose ≥126 mg/dL or hemoglobin A1_C_ ≥o6.5% or current intake of anti-diabetes medications. Dyslipidemia was defined as either total cholesterol ≥240 mg/dL or the current use of anti-dyslipidemia drugs.

Study protocol was approved by institutional review board of each participating hospital and all patients gave written informed consent. A total of 2960 patients were registered consecutively from May 2010 to June 2015 in 11 tertiary hospitals in Korea with high volume CAG and percutaneous coronary intervention (PCI) performances. Among them, 257 had missing data and 2703 were included in analysis. A total of 1836 patients were diagnosed to have positive results in their provocation tests 867 had negative results (Fig. 1). Each spastic lesion, 881 in LAD, 419 in LCx, 942 in RCA were included in lesion specific analysis (Fig. 1).

### Coronary angiography and EG provocation test

We followed provocation methods from Japanese Circulation Society guideline for diagnosis and treatment of patients with VA^27^. Detailed methods of CAG, EG provocation test and adjudication of EG provocation test results are reported previously^25,26^.

Quantitative coronary angiography (QCA) was performed twice, firstly in each responsible hospital by the personnel who are not aware of the study and secondly by the dedicated physicians who were blinded to the study at core laboratory located in Seoul St. Mary’s Hospital using dedicated QCA program and by visual assessment.

### Coronary plaque analysis

Firstly, we compared the overall plaque status of coronary arteries like distribution, frequency and % diameter stenosis between VA patients and non-VA patients. Baseline characteristics between VA and non-VA patients were compared (Table 1).

Secondly, we focused on each spastic coronary artery. We compared plaque status (including plaque frequency, mean diameter % stenosis, total plaque burden) between spastic artery and non-spastic artery in same VA-patient. We also compared spasm positive arteries of VA-patients with index arteries which are negative spasm but other site spasm positive one [INOP] or non-VA patients regarding to plaque status. We also compared spasm related index artery and non-spasm related artery with corresponding arteries of non-VA patient.

In this spasm related coronary focused analysis, we only included patients with coronary diameter of ≥2.5 mm artery which had available data on spasm location (location mean name of coronary like LAD, LCx, RCA). Patients having multi-vessel spasm are included several times for each arterial analysis (Fig. 1). For example, if a patient had both LAD and LCx spasm, their atherosclerosis on LAD and LCx were both analyzed as individual coronary artery. Thus the LAD spasm 881, LCx 419, RCA 942 lesions were included in analysis. Overlapped spasm positive coronary arteries ranging from 2 to 4 times were described in detail in Fig. 1 study flow.

Thirdly, we are also pursue to find the atherosclerotic % stenosis which could induce vasospasm with sensitivity and specificity test starting form 30% until 70% step by step with 5% increment of diameter stenosis in each coronary artery by comparing diameter % stenosis between index arterial spasm positive patients and index arterial spasm negative patients (which included INOP and non-VA patients). We also tested this cut-off value of each coronary artery and composite cut-off values of LAD or LCx or RCA by multivariate logistic regression analysis.

Coronary percent diameter stenosis was estimated by visual estimation and QCA in coronary angiography in central lab. Plaque was defined as ≥1% luminal narrowing of coronary diameter. If a coronary artery had at least one plaque in any segment of the same coronary artery, that was defined as having a plaque. If at least one plaque was present among 3 coronary arteries, a patient was regarded to have plaque. Mean % diameter stenosis of each coronary artery was calculated by averaging each % stenosis of proximal, medial and distal segment. Plaque burden was defined as summation of % stenosis of each segment, i.e. the maximum 300 and minimum 1. We do not have intravascular ultrasound (IVUS) data and could not exactly estimate the plaque volume, thus we adopt this indirect methods of plaque summation.

### Statistical analysis

Continuous variables are expressed as mean ± standard deviation and categorical variables are presented as frequencies and percentages. For between-group comparisons, independent t-test or Mann-Whitney U-test were used for continuous variables, and the chi-square test or Fisher’s exact test were used for categorical variables. A simple logistic regression and multivariate logistic regression model were used to identify independent predictors of cut-off values of diameter percent stenosis for determining vasospasm.

We performed backward stepwise logistic regression analysis using variables in Table 1 in testing the each variable of LAD ≥ 35%, LCx ≥ 35%, and RCA ≥ 40% as independent risk factors for inducing spasm respectively. We also conducted logistic regression analysis with same manner in testing composite of LAD ≥ 35% or LCx ≥ 35% or RCA ≥ 40% as independent risk of provoking spasm. Gender, age, diabetes and LAD ≥ 35% were significant in testing LAD ≥ 35%. Gender, dyslipidemia and LCx ≥ 35% were also significant in LCx ≥ 35%. Gender, age, diabetes and smoking were found to be significant in testing RCA ≥ 40%. The above variables and the variables that investigator considered being important such as age, hypertension, diabetes, smoking, history of using calcium channel blockers were included in the multiple logistic regression analysis as fixed variables. Statistical analysis was carried out by using SPSS ver. 23 (IBM Corporation).

## Conclusions

VA patient has more probability of having atherosclerotic plaque, higher lumen stenosis and larger plaque burden even in non-spasm related coronary artery than in non-VA patients. Atherosclerotic lumen narrowing of ≥35–40% in coronary artery could be a cut-off value for development coronary vasospasm.

## Supplementary information

Supplementary tables and figures.
