# Supplementary material for: Coronary Plaque Characteristics and Cut-Off Stenosis for Developing Spasm in Patients with Vasospastic Angina
Source: Sci Rep. 2020 Mar 31;10:5707. doi: 10.1038/s41598-020-62670-z (PMC7109107; doi:10.1038/s41598-020-62670-z)
Supplement: Supplementary file 1 — Supplementary tables and figures. [file 41598_2020_62670_MOESM1_ESM.docx]

**Coronary Plaque Characteristics and Cut-Off Stenosis for Developing Spasm in Patients with Vasospastic Angina**

Sang-Ho Jo, MD^a*^; Ju Ho Sim, MS^b^; Sang Hong Baek, MD^c^

^a^ Division of Cardiology, Department of Internal Medicine, Hallym University Sacred Heart Hospital, Anyang-si, Gyeonggi-do, Korea

^b^ Graduate School of Public Health, Yonsei University, Seoul, South Korea

^c^ Department of Cardiovascular Medicine, Seoul St. Mary’s Hospital, The Catholic University

of Korea, Seoul, South Korea

**Supplementary Tables 1.** Plaque frequency, mean diameter stenosis and plaque burden of all coronary arteries stratified by spasm positivity at index coronary vs. index negative but other site positive vs. non-VA patients

1. **LAD**

|  | LAD Spasm Positive ^a^ (N=881)  n (%) | LAD spasm negative,  Other site spasm positive ^b^ (N=954)  n (%) | Non-VA ^c^ (N=867)  n (%) | p-value |
| --- | --- | --- | --- | --- |
| **LAD plaque Yes** | **243 (27.6)** | **156 (16.4)** | **109 (12.6)** | **<0.001** |
| LAD plaque No | 638 (72.4) | 798 (83.6) | 758 (87.4) |  |
|  | LAD Spasm Positive ^a^ (N=881)  n (%) | LAD spasm negative,  Other site spasm positive ^b^ (N=954)  n (%) | Non-VA ^c^ (N=867)  n (%) | p-value |
| LCx plaque Yes | 71 (8.1) | 94 (9.9) | 28 (3.2) | <0.001 |
| LCx plaque No | 810 (91.9) | 860 (90.1) | 839 (96.8) |  |
|  | LAD Spasm Positive ^a^ (N=881)  n (%) | LAD spasm negative,  Other site spasm positive ^b^ (N=954)  n (%) | Non-VA ^c (^N=867)  n (%) | p-value |
| RCA plaque Yes | 90 (10.2) | 211 (22.1) | 57 (6.6) | <0.001 |
| RCA plaque No | 791 (89.8) | 743 (77.9) | 810 (93.4) |  |

|  | LAD Spasm Positive ^a^ (N=243) | LAD spasm negative,  Other site spasm positive ^b^ (N=156) | Non-VA ^c^ (N=109) | p-value |
| --- | --- | --- | --- | --- |
| LAD stenosis (%) | 38.3±16.5 | 34.0±16.5 | 32.4±14.2 | 0.002^a>bc^ |
|  |  |  |  |  |
|  | LAD Spasm Positive ^a^ (N=71) | LAD spasm negative,  Other site spasm positive ^b^ (N=94) | Non-VA ^c^ (N=28) | p-value |
| LCx stenosis (%) | 32.5±13.2 | 38.8±21.1 | 28.0±8.2 | 0.005 ^b>ac^ |
|  |  |  |  |  |
|  | LAD Spasm Positive ^a^ (N=90) | LAD spasm negative,  Other site spasm positive ^b^ (N=211) | Non-VA ^c^ (N=57) | p-value |
| RCA stenosis (%) | 33.2±17.4 | 36.1±17.6 | 30.2±15.5 | 0.056 |

|  | LAD Spasm Positive ^a^  (N=243) | LAD spasm negative,  Other site spasm positive ^b^  (N=156) | Non-VA ^c^  (N=109) | p-value |
| --- | --- | --- | --- | --- |
| LAD stenosis summation | 45.2±23.2 | 42.1±30.8 | 38.4±28.9 | 0.090 |
|  |  |  |  |  |
|  | LAD Spasm Positive ^a^  (N=71) | LAD spasm negative,  Other site spasm positive ^b^  (N=94) | Non-VA ^c^  (N=28) | p-value |
| LCx stenosis summation | 39.8±21.6 | 42.0±22.0 | 31.3±15.4 | 0.063 |
|  |  |  |  |  |
|  | LAD Spasm Positive ^a^  (N=90) | LAD spasm negative,  Other site spasm positive ^b^  (N=211) | Non-VA ^c^  (N=57) | p-value |
| RCA stenosis summation | 39.9±23.6 | 43.1±26.1 | 37.2±31.2 | 0.274 |

1. **LCX**

|  | LCx Spasm Positive ^a^ (N=419)  n (%) | LCx spasm negative,  Other site spasm positive ^b^ (N=1,417)  n (%) | Non-VA ^c^ (N=867)  n (%) | p-value |
| --- | --- | --- | --- | --- |
| LAD plaque Yes | 73 (17.4) | 326 (23.0) | 109 (12.6) | <0.001 |
| LAD plaque No | 346 (82.6) | 1,091 (77.0) | 758 (87.2) |  |
|  | LCx Spasm Positive ^a^ (N=419)  n (%) | LCx spasm negative,  Other site spasm positive ^b^ (N=1,417)  n (%) | Non-VA ^c^ (N=867)  n (%) | p-value |
| LCx plaque Yes | 68 (16.2) | 97 (6.8) | 28 (3.2) | <0.001 |
| LCx plaque No | 351 (83.8) | 1,320 (93.2) | 839 (96.8) |  |
|  | LCx Spasm Positive ^a^ (N=419)  n (%) | LCx spasm negative,  Other site spasm positive ^b^ (N=1,417)  n (%) | Non-VA ^c^ (N=867)  n (%) | p-value |
| RCA plaque Yes | 46 (11.0) | 255 (18.0) | 57 (6.6) | <0.001 |
| RCA plaque No | 373 (89.0) | 1,162 (82.0) | 812 (93.7) |  |

|  | LCx Spasm Positive^a^  (N=73) | LCx spasm negative,  Other site spasm positive ^b^  (N=326) | Non-VA ^c^  (N=109) | p-value |
| --- | --- | --- | --- | --- |
| LAD stenosis (%) | 36.0±15.0 | 36.7±17.0 | 32.4±14.2 | 0.059 |
|  |  |  |  |  |
|  | LCx Spasm Positive ^a^  (N=68) | LCx spasm negative,  Other site spasm positive ^b^  (N=97) | Non-VA ^c^  (N=28) | p-value |
| LCx stenosis (%) | 40.2±19.6 | 33.2±16.9 | 28.0±8.2 | 0.003 ^a>bc^ |
|  |  |  |  |  |
|  | LCx Spasm Positive ^a^  (N=46) | LCx spasm negative,  Other site spasm positive ^b^  (N=255) | Non-VA ^c^  (N=57) | p-value |
| RCA stenosis (%) | 35.3±18.5 | 35.2±17.4 | 30.2±15.5 | 0.140 |

|  | LCx Spasm Positive ^a^  (N=73) | LCx spasm negative,  Other site spasm positive ^b^  (N=326) | Non-VA ^c^  (N=109) | p-value |
| --- | --- | --- | --- | --- |
| LAD stenosis summation | 45.1±26.7 | 43.8±26.4 | 38.4±28.9 | 0.157 |
|  |  |  |  |  |
|  | LCx Spasm Positive ^a^  (N=68) | LCx spasm negative,  Other site spasm positive ^b^  (N=97) | Non-VA ^c^  (N=28) | p-value |
| LCx stenosis summation | 44.8±20.6 | 38.4±22.3 | 31.3±15.4 | 0.012 ^a>c^ |
|  |  |  |  |  |
|  | LCx Spasm Positive ^a^  (N=46) | LCx spasm negative,  Other site spasm positive ^b^  (N=255) | Non-VA ^c^  (N=57) | p-value |
| RCA stenosis summation | 46.4±35.9 | 41.4±23.0 | 37.2±31.2 | 0.217 |

1. **RCA**

|  | RCA Spasm Positive ^a^ (N=942)  n (%) | RCA spasm negative,  Other site spasm positive ^b^ (N=893)  n (%) | Non-VA ^c^ (N=867)  n (%) | p-value |
| --- | --- | --- | --- | --- |
| LAD plaque Yes | 175 (18.6) | 224 (25.1) | 109 (12.6) | <0.001 |
| LAD plaque No | 767 (81.4) | 669 (74.9) | 758(87.4) |  |
|  | RCA Spasm Positive ^a^ (N=942)  n (%) | RCA spasm negative,  Other site spasm positive ^b^ (N=893)  n (%) | Non-VA ^c^ (N=867)  n (%) | p-value |
| LCx plaque Yes | 74 (7.9) | 91 (10.2) | 28 (3.2) | <0.001 |
| LCx plaque No | 868 (92.1) | 802 (89.8) | 839 (96.8) |  |
|  | RCA Spasm Positive ^a^ (N=942)  n (%) | RCA spasm negative,  Other site spasm positive ^b^ (N=802)  n (%) | Non-VA ^c^ (N=867)  n (%) | p-value |
| RCA plaque Yes | 213 (22.6) | 82 (10.2) | 57 (6.6) | <0.001 |
| RCA plaque No | 729 (77.4) | 720 (89.8) | 812 (93.7) |  |

|  | RCA Spasm Positive ^a^  (N=175) | RCA spasm negative,  Other site spasm positive ^b^  (N=224) | Non-VA ^c^  (N=109) | p-value |
| --- | --- | --- | --- | --- |
| LAD stenosis (%) | 33.9±16.4 | 38.7±16.6 | 32.4±14.2 | 0.001 ^b>ac^ |
|  |  |  |  |  |
|  | RCA Spasm Positive ^a^  (N=74) | RCA spasm negative,  Other site spasm positive ^b^  (N=91) | Non-VA ^c^  (N=28) | p-value |
| LCx stenosis (%) | 32.0±14.1 | 39.4±20.6 | 28.0±8.2 | 0.002 ^b>ac^ |
|  |  |  |  |  |
|  | RCA Spasm Positive ^a^  (N=213) | RCA spasm negative,  Other site spasm positive ^b^  (N=88) | Non-VA ^c^  (N=57) |  |
| RCA stenosis (%) | 35.8±17.8 | 33.8±16.8 | 30.2±15.5 | 0.092 |

|  | RCA Spasm Positive ^a^  (N=175) | RCA spasm negative,  Other site spasm positive ^b^  (N=224) | Non-VA ^c^  (N=109) | p-value |
| --- | --- | --- | --- | --- |
| LAD stenosis summation | 40.9±27.3 | 46.5±25.6 | 38.4±28.9 | 0.021 ^b>ac^ |
|  |  |  |  |  |
|  | RCA Spasm Positive ^a^  (N=74) | RCA spasm negative,  Other site spasm positive ^b^  (N=91) | Non-VA ^c^  (N=28) | p-value |
| LCx stenosis summation | 35.0±16.8 | 45.9±24.1 | 31.3±15.4 | <0.001 ^b>ac^ |
|  |  |  |  |  |
|  | RCA Spasm Positive ^a^  (N=213) | RCA spasm negative,  Other site spasm positive ^b^  (N=88) | Non-VA ^c^  (N=57) |  |
| RCA stenosis summation | 42.1±23.8 | 42.2±28.9 | 37.2±31.2 | 0.439 |

LAD, left anterior descending coronary artery; LCx, left circumflex coronary artery; RCA, right coronary artery

**Supplementary Tables 2.** Determining diameter stenosis cut-off value for predicting vasospasm comparing with non-spasm coronary at index coronary (including index negative other site positive coronary and non-VA coronary) by sensitivity and specificity test with increment of 5% diameter stenosis from 30% to 70% in each coronary artery

LAD$\geq$35%, LCx$\geq$35%, and RCA$\geq$40% coronary luminal stenosis were appropriate for determining vasospasm with adequate sensitivity and specificity.

(A) LAD

| Cut-Off % stenosis | Total | LAD spasm Positive  (N=243) | LAD spasm Negative  (N=262) | p-value | OR | 95%CI | p-value |
| --- | --- | --- | --- | --- | --- | --- | --- |
| $\geq$30% | 371(73.5) | 193(79.4) | 178(67.9) | 0.003 | 1.822 | 1.215-2.731 | 0.004 |
| <30% | 134(26.5) | 50(20.6) | 84(32.1) |  | 1 |  |  |
|  | Total | LAD spasm Positive  (N=243) | LAD spasm Negative  (N=262) | p-value | OR | 95%CI | p-value |
| $\boldsymbol{\geq}$**35%** | 204(40.4) | **116(47.7)** | 88(33.6) | **0.001** | **1.806** | **1.261-2.587** | **0.001** |
| **<35%** | 301(59.6) | 127(52.3) | **174(66.4)** |  | 1 |  |  |
|  | Total | LAD spasm Positive  (N=243) | LAD spasm Negative  (N=262) | p-value | OR | 95%CI | p-value |
| $\geq$40% | 193(38.2) | 111(45.7) | 82(31.3) | 0.001 | 1.846 | 1.284-2.654 | 0.001 |
| <40% | 312(61.8) | 132(54.3) | 180(68.7) |  | 1 |  |  |
|  | Total | LAD spasm Positive  (N=243) | LAD spasm Negative  (N=262) | p-value | OR | 95%CI | p-value |
| $\geq$45% | 128(25.3) | 74(30.5) | 54(20.6) | 0.011 | 1.687 | 1.125-2.529 | 0.011 |
| <45% | 377(74.7) | 169(69.5) | 208(79.4) |  | 1 |  |  |
|  | Total | LAD spasm Positive  (N=243) | LAD spasm Negative (N=262) | p-value | OR | 95%CI | p-value |
| $\geq$50% | 122(24.2) | 71(29.2) | 51(19.5) | 0.011 | 1.708 | 1.131-2.579 | 0.011 |
| <50% | 383(75.8) | 172(70.8) | 211(80.5) |  | 1 |  |  |
|  | Total | LAD spasm Positive  (N=243) | LAD spasm Negative (N=262) | p-value | OR | 95%CI | p-value |
| $\geq$55% | 52(10.3) | 34(14.0) | 18(6.9) | 0.009 | 2.205 | 1.210-4.020 | 0.010 |
| <55% | 453(89.7) | 209(86.0) | 244(93.1) |  | 1 |  |  |
|  | Total | LAD spasm Positive  (N=243) | LAD spasm Negative (N=262) | p-value | OR | 95%CI | p-value |
| $\geq$60% | 48(9.5) | 31(12.8) | 17(6.5) | 0.016 | 2.107 | 1.134-3.915 | 0.018 |
| <60% | 457(90.5) | 212(87.2) | 245(93.5) |  | 1 |  |  |
|  | Total | LAD spasm Positive  (N=243) | LAD spasm Negative  (N=262) | p-value | OR | 95%CI | p-value |
| $\geq$65% | 52(10.3) | 34(14.0) | 18(6.9) | 0.009 | 1.607 | 0.828-3.121 | 0.161 |
| <65% | 453(89.7) | 209(86.0) | 244(93.1) |  | 1 |  |  |
|  | Total | LAD spasm Positive  (N=243) | LAD spasm Negative  (N=262) | p-value | OR | 95%CI | p-value |
| $\geq$70% | 37(7.3) | 22(9.1) | 15(5.7) | 0.152 | 1.639 | 0.830-3.239 | 0.155 |
| <70% | 468(92.7) | 221(90.9) | 247(94.3) |  | 1 |  |  |

(B) LCx

|  | Total | LCX spasm Positive  (N=68) | LCX spasm Negative  (N=125) | p-value | OR | 95%CI | p-value |
| --- | --- | --- | --- | --- | --- | --- | --- |
| $\geq$30% | 138(71.5) | 53(77.9) | 85(68.0) | 0.144 | 1.663 | 0.838-3.300 | 0.146 |
| <30% | 55(28.5) | 15(22.1) | 40(32.0) |  | 1 |  |  |
|  | Total | LCX spasm Positive  (N=68) | LCX spasm Negative  (N=125) | p-value | OR | 95%CI | p-value |
| $\boldsymbol{\geq}$**35%** | 72(37.3) | **36(52.9)** | 36(28.8) | **0.001** | **2.781** | **1.506-5.138** | **0.001** |
| **<35%** | 121(62.7) | 32(47.1) | **89(71.2)** |  | 1 |  |  |
|  | Total | LCX spasm Positive  (N=68) | LCX spasm Negative (N=125) | p-value | OR | 95%CI | p-value |
| $\geq4$0% | 66(34.2) | 34(50.0) | 32(25.6) | 0.001 | 2.906 | 1.560-5.415 | 0.001 |
| <40% | 127(65.8) | 34(50.0) | 93(74.4) |  | 1 |  |  |
|  | Total | LCX spasm Positive  (N=68) | LCX spasm Negative  (N=125) | p-value | OR | 95%CI | p-value |
| $\geq$45% | 36(18.7) | 19(27.9) | 17(13.6) | 0.015 | 2.463 | 1.180-5.144 | 0.016 |
| <45% | 157(81.3) | 49(72.1) | 108(86.4) |  | 1 |  |  |
|  | Total | LCX spasm Positive  (N=68) | LCX spasm Negative  (N=125) | p-value | OR | 95%CI | p-value |
| $\geq$50% | 35(18.1) | 19(27.9) | 16(12.8) | 0.009 | 2.642 | 1.253-5.568 | 0.011 |
| <50% | 158(81.9) | 49(72.1) | 109(87.2) |  | 1 |  |  |
|  | Total | LCX spasm Positive (N=68) | LCX spasm Negative  (N=125) | p-value | OR | 95%CI | p-value |
| $\geq$55% | 19(9.8) | 12(17.6) | 7(5.6) | 0.007 | 3.612 | 1.349-9.672 | 0.011 |
| <55% | 174(90.2) | 56(82.4) | 118(94.4) |  | 1 |  |  |
|  | Total | LCX spasm Positive  (N=68) | LCX spasm Negative  (N=125) | p-value | OR | 95%CI | p-value |
| $\geq$60% | 19(9.8) | 12(17.6) | 7(5.6) | 0.007 | 3.612 | 1.349-9.672 | 0.011 |
| <60% | 174(90.2) | 56(82.4) | 118(94.4) |  | 1 |  |  |
|  | Total | LCX spasm Positive  (N=68) | LCX spasm Negative (N=125) | p-value | OR | 95%CI | p-value |
| $\geq$65% | 13(6.7) | 7(10.3) | 6(4.8) | 0.227 | 2.276 | 0.733-7.069 | 0.155 |
| 65% | 180(93.3) | 61(89.7) | 119(95.2) |  | 1 |  |  |
|  | Total | LCX spasm Positive  (N=68) | LCX spasm Negative (N=125) | p-value | OR | 95%CI | p-value |
| $\geq$70% | 12(6.2) | 6(8.8) | 6(4.8) | 0.350 | 1.919 | 0.594-6.200 | 0.276 |
| <70% | 181(93.8) | 62(91.2) | 119(95.2) |  | 1 |  |  |

(C) RCA

|  | Total | RCA spasm Positive  (N=213) | RCA spasm Negative (N=143) | p-value | OR | 95%CI | p-value |
| --- | --- | --- | --- | --- | --- | --- | --- |
| $\geq$30% | 239(67.1) | 148(69.5) | 91(63.6) | 0.250 | 1.301 | 0.831-2.037 | 0.250 |
| <30% | 117(32.9) | 65(30.5) | 52(36.4) |  | 1 |  |  |
|  | Total | RCA spasm Positive  (N=213) | RCA spasm Negative  (N=143) | p-value | OR | 95%CI | p-value |
| $\geq$35% | 121(34.0) | 80(37.6) | 41(28.7) | 0.083 | 1.496 | 0.948-2.362 | 0.083 |
| <35% | 235(66.0) | 133(62.4) | 102(71.3) |  | 1 |  |  |
|  | Total | RCA spasm Positive (N=213) | RCA spasm Negative (N=143) | p-value | OR | 95%CI | p-value |
| $\boldsymbol{\geq}$**40%** | 116(32.6) | **78(36.6)** | 38(26.6) | **0.047** | **1.596** | **1.004-2.539** | **0.048** |
| **<40%** | 240(67.4) | 135(63.4) | **105(73.4)** |  | 1 |  |  |
|  | Total | RCA spasm Positive  (N=213) | RCA spasm Negative  (N=143) | p-value | OR | 95%CI | p-value |
| $\geq$45% | 78(21.9) | 55(25.8) | 23(16.1) | 0.029 | 1.816 | 1.057-3.121 | 0.031 |
| <45% | 278(78.1) | 158(74.2) | 120(83.9) |  | 1 |  |  |
|  | Total | RCA spasm Positive  (N=213) | RCA spasm Negative  (N=143) | p-value | OR | 95%CI | p-value |
| $\geq$50% | 74(20.8) | 51(23.9) | 23(16.1) | 0.073 | 1.643 | 0.951-2.835 | 0.075 |
| <50% | 282(79.2) | 162(76.1) | 120(83.9) |  | 1 |  |  |
|  | Total | RCA spasm Positive  (N=213) | RCA spasm Negative  (N=143) | p-value | OR | 95%CI | p-value |
| $\geq$55% | 36(10.1) | 25(11.7) | 11(7.7) | 0.215 | 1.596 | 0.759-3.355 | 0.218 |
| <55% | 320(89.9) | 188(88.3) | 132(92.3) |  | 1 |  |  |
|  | Total | RCA spasm Positive  (N=213) | RCA spasm Negative  (N=143) | p-value | OR | 95%CI | p-value |
| $\geq$60% | 36(10.1) | 25(11.7) | 11(7.7) | 0.215 | 1.596 | 0.759-3.355 | 0.218 |
| <60% | 320(89.9) | 188(88.3) | 132(92.3) |  | 1 |  |  |
|  | Total | RCA spasm Positive  (N=213) | RCA spasm Negative  (N=143) | p-value | OR | 95%CI | p-value |
| $\geq$65% | 25(7.0) | 16(7.5) | 9(6.3) | 0.659 | 1.209 | 0.519-2.817 | 0.660 |
| <65% | 331(93.0) | 197(92.5) | 134(93.7) |  | 1 |  |  |
|  | Total | RCA spasm Positive  (N=213) | RCA spasm Negative  (N=143) | p-value | OR | 95%CI | p-value |
| $\geq$70% | 25(7.0) | 16(7.5) | 9(6.3) | 0.659 | 1.209 | 0.519-2.817 | 0.660 |
| <70% | 331(93.0) | 197(92.5) | 134(93.7) |  | 1 |  |  |

LAD, left anterior descending coronary artery; LCx, left circumflex coronary artery; RCA, right coronary artery

Supplementary Table 3. Multivariate logistic regression analysis on each coronary artery stenosis determining spasm positivity

1. **LAD**

|  |  | Spasm Negative  (N=867) | Spasm Positive  (N=881) | P-value | OR | 95% CI | P-value |
| --- | --- | --- | --- | --- | --- | --- | --- |
| Gender | Female | 531 (61.2) | 367 (41.7) | <0.001 | 1 |  |  |
|  | male | 336 (38.8) | 514 (58.1) |  | 2.455 | 1.376-4.3779 | **0.002** |
| Age |  | 54.7±13.0 | 53.6±11.7 | 0.046 | 0.974 | 0.950-0.998 | **0.037** |
| BMI(kg/m2) |  | 24.7±3.4 | 24.7±3.2 | 0.948 | 0.955 | 0.879-1.038 | 0.279 |
| Hypertension | No | 560 (64.6) | 574 (65.2) | 0.780 | 1 |  |  |
|  | Yes | 307 (35.4) | 306 (34.8) |  | 1.226 | 0.667-2.253 | 0.512 |
| Diabetes | No | 781 (90.1) | 786 (89.5) | 0.700 | 1 |  |  |
|  | Yes | 86 (9.9) | 92 (10.5) |  | 0.626 | 0.301-1.303 | 0.210 |
| Dyslipidemia | No | 722 (83.3) | 744 (85.0) | 0.316 | 1 |  |  |
|  | Yes | 145 (16.7) | 131 (15.0) |  | 0.572 | 0.276-1.184 | 0.132 |
| Smoking* | No | 738 (86.2) | 632 (72.6) | <0.001 | 1 |  |  |
|  | Yes | 118 (13.8) | 239 (27.4) |  | 2.493 | 1.067-5.822 | **0.035** |
| Hx CCB | No | 703 (81.5) | 719 (83.0) | 0.394 | 1 |  |  |
|  | Yes | 160 (18.5) | 147 (17.0) |  | 0.563 | 0.287-1.103 | 0.094 |
| LAD stenosis | Under 35% | 75 (70.8) | 127 (52.3) | 0.001 | 1 |  |  |
|  | Over 35% | 31 (29.2) | 116 (47.7) |  | 2.434 | 1.400-4.232 | **0.002** |

*no: never & Ex-smoker / Yes: Active-smoker

1. LCx

|  |  | Spasm Negative  (N=867) | Spasm Positive  (N=419) | P-value | OR | 95% CI | P-value |
| --- | --- | --- | --- | --- | --- | --- | --- |
| Gender | Female | 531 (61.2) | 176 (42.0) | <0.001 | 1 |  |  |
|  | male | 336 (38.8) | 243 (58.0) |  | 3.756 | 1.057-13.347 | **0.041** |
| Age |  | 54.7±13.0 | 56.2±11.3 | 0.045 | 1.028 | 0.974-1.084 | 0.318 |
| BMI(kg/m2) |  | 24.7±3.4 | 25.1±3.3 | 0.067 | 1.117 | 0.900-1.387 | 0.315 |
| Hypertension | No | 560 (64.6) | 249 (59.6) | 0.081 | 1 |  |  |
|  | Yes | 307 (35.4) | 169 (40.4) |  | 0.509 | 0.154-1.681 | 0.268 |
| Diabetes | No | 781 (90.1) | 372 (89.2) | 0.629 | 1 |  |  |
|  | Yes | 86 (9.9) | 45 (10.8) |  | 0.783 | 0.197-3.117 | 0.728 |
| Dyslipidemia | No | 722 (83.3) | 342 (82.2) | 0.635 | 1 |  |  |
|  | Yes | 145 (16.7) | 74 (17.8) |  | 0.346 | 0.085-1.419 | 0.141 |
| Smoking* | No | 738 (86.2) | 298 (72.5) | <0.001 | 1 |  |  |
|  | Yes | 118 (13.8) | 113 (27.5) |  | 2.971 | 0.459-19.239 | 0.253 |
| Hx CCB | No | 703 (81.5) | 321 (77.5) | 0.100 | 1 |  |  |
|  | Yes | 160 (18.5) | 93 (22.5) |  | 1.080 | 0.324-3.600 | 0.900 |
| LCx stenosis | Under 35% | 25 (89.3) | 32 (47.1) | <0.001 | 1 |  |  |
|  | Over 35% | 3 (10.7) | 36 (52.9) |  | 8.349 | 2.086-33.412 | **0.003** |

*no: never & Ex-smoker / Yes: Active-smoker

(C) RCA

|  |  | Spasm Negative (N=867) | Spasm Positive  (N=942) | P-value | OR | 95% CI | P-value |
| --- | --- | --- | --- | --- | --- | --- | --- |
| Gender | Female | 531 (61.2) | 344 (36.5) | <0.001 | 1 |  |  |
|  | male | 336 (33.8) | 598 (63.5) |  | 3.235 | 1.547-6.762 | **0.002** |
| Age |  | 54.7±13.0 | 55.8±11.0 | 0.063 | 1.017 | 0.980-1.055 | 0.377 |
| BMI(kg/m2) |  | 24.7±3.4 | 24.8±3.9 | 0.643 | 1.023 | 0.943-1.110 | 0.586 |
| Hypertension | No | 560 (64.6) | 565 (60.0) | 0.043 | 1 |  |  |
|  | Yes | 307 (35.4) | 377 (40.0) |  | 1.332 | 0.645-2.753 | 0.439 |
| Diabetes | No | 781 (90.1) | 867 (92.0) | 0.144 | 1 |  |  |
|  | Yes | 86 (9.9) | 75 (8.0) |  | 0.312 | 0.118-0.825 | **0.019** |
| Dyslipidemia | No | 722 (83.3) | 773 (82.1) | 0.526 | 1 |  |  |
|  | Yes | 145 (16.7) | 168 (17.9) |  | 569 | 0.243-1.336 | 0.196 |
| Smoking* | No | 738 (86.2) | 673 (72.1) | <0.001 | 1 |  |  |
|  | Yes | 118 (13.8) | 261 (27.9) |  | 2.697 | 0.902-8.070 | 0.076 |
| Hx CCBs | No | 703 (81.5) | 731 (78.3) | 0.101 | 1 |  |  |
|  | Yes | 160 (18.5) | 202 (21.7) |  | 0.489 | 0.234-1.024 | 0.058 |
| RCA stenosis | Under 40% | 40 (72.7) | 135 (63.4) | 0.194 | 1 |  |  |
|  | Over 40% | 15 (27.3) | 78 (36.6) |  | 1.647 | 0.781-3.472 | 0.190 |

*no: never & Ex-smoker / Yes: Active-smoker

OR, odds ratio; CI, confidence interval; BMI, body mass index; Hx, history; CCBs, calcium channel blockers; LAD, left anterior descending coronary artery; LCx, left circumflex coronary artery; RCA, right coronary artery

**Supplementary Figure 1.** Distribution of spasm positive arteries and multiple spasm artery combination

**Supplementary Figure 2.** Atherosclerotic diameter percent stenosis according to spasm positivity on each index coronary artery, index coronary spasm negative and other coronary spasm positive (INOP) and spasm negative patient

1. LAD
2. LCx
3. RCA

LAD, left anterior descending coronary artery; LCx, left circumflex coronary artery; RCA, right coronary artery
